# The Presence and Nature of AI-Use Disclosure Statements in Medical Education Journals: A Bibliometric Study

**DOI:** 10.5334/pme.2431

**Published:** 2026-03-05

**Authors:** Muhammad Ans, Lauren A. Maggio, Hamzah Algodi, Joe A. Costello, Erik Driessen, Kevin Oswald, Lorelei Lingard

**Affiliations:** 1 Schulich School of Medicine & Dentistry, Western University, CA; 2 Department of Medical Education, University of Illinois Chicago, US; 3 Faculty of Health, Medicine & Life Sciences, Maastricht University, NL; 4 Faculty of Information & Media Studies, Western University, CA

## Abstract

**Background:**

As AI use becomes more common in research, disclosure policies have emerged to ensure transparency and appropriateness. However, database research in other fields suggests that disclosure may lag behind AI use. Medical education journal editors report that submitted manuscripts rarely include AI-use disclosures, and they perceive a lack of clarity regarding when and how AI use should be disclosed. However, we lack objective evidence regarding the incidence and nature of AI-use disclosure in medical education.

**Methods:**

Using bibliometric methods, we searched a database of 24 leading medical education journals for articles published between January and July 2025 (n = 2,762 articles). Screening with *Covidence* software excluded 716 non-empirical and/or non-English language articles. The remainder (n = 2,046) were examined for the presence of AI-use disclosures, which were content-analyzed.

**Results:**

2.5% of empirical articles (n = 51) had an AI disclosure statement. *BMC Medical Education* contained the most disclosures (24), followed by *Medical Teacher* (7) and *Journal of Surgical Education* (4). Forty-two articles were authored in non-native English-speaking countries, and 69.4% of all first authors had begun publishing in the past decade. Disclosures averaged 43 words and described use superficially: most commonly “editing” and “translation”. Of 18 named tools, ChatGPT was most common. Most disclosures explicitly attested to author responsibility for AI-produced material. Disclosures usually appeared in acknowledgements; those located in methods lacked responsibility attestation. Negative disclosures attesting that AI was not used were also present.

**Discussion:**

AI-use disclosures in medical education journals are rare and appear mostly in work from non-native English-speaking regions of the world. A shared disclosure practice is evident: name the tool and affirm author responsibility, but describe use superficially. This suggests a practice of “safe” disclosure that may be more performative than informative, therefore failing to satisfy the goal of ensuring transparent and ethical AI use in research.

## Background

AI has the potential to transform research practices by enhancing productivity in both research and writing. At the same time, its use introduces significant ethical and methodological challenges. When applied uncritically or without transparent reporting, AI may compromise research integrity through issues such as unclear attribution of intellectual contribution or obscured methodological decisions [1,2]. Consequently, disclosure policies from organizations such as the International Committee of Medical Journal Editors [3], publishers and journals [4,5] share a general emphasis on transparency and accountability. While specific requirements vary, authors are required to explicitly disclose whether they used AI-assisted technologies in the production of submitted work, and to take responsibility for all AI-produced material [6,7].

However, emerging research suggests that AI disclosure may be lagging behind AI use. A 2025 Nature survey of 5000 researchers found diverging views of disclosure [8]. For instance, when asked whether they had used AI to write a section of a paper and not disclosed the AI use, 17% of mid-career researchers said ‘Yes’, and 47% said ‘No, but I would be willing to’. Database research also suggests that AI use outstrips disclosure. For instance, one comparative study of >5 million articles in the Dimensions database reported a 468% increase in clusters of ChatGPT-preferred positive terms (e.g., meticulous, intricate, commendable) in texts published in 2023 after the model became available; only 0.1% of those papers contained language suggesting disclosure [9]. Similarly, a 2024 analysis of abstracts published in high-impact orthopaedic journals found that of 28 containing AI-generated text, only 1 disclosed AI use abuzz with keynotes, panels, workshops, and papers related to AI [10]. But while it is increasingly apparent that scholars are using AI [11], the nature and pattern of our disclosure practices are less clear. In a recent interview study, medical education journal editors described infrequent experience with AI-use disclosures in submitted manuscripts [12]. Most suspected that medical education researchers might be using AI without disclosing, and worried that this may arise from a lack of clarity regarding when disclosure is necessary and what details are required.

To deepen understanding of AI-use disclosure in medical education, we require objective evidence of researchers’ current disclosure practices. Therefore, this study asks: What is the rate and nature of AI-use disclosure in empirical research papers in medical education journals?

## Methods

We conducted a bibliometric study to identify and characterize AI disclosure statements appearing in medical education research articles. Our sampling frame included all articles published between January 1 and June 30, 2025, in the 24 leading medical education journals listed in the Medical Education Journals List, which is a set of journals created via bibliometric methods to identify core medical education journals that serve as central outlets for scholarship in the field [13]. We selected a 6-month sampling period to balance feasibility with adequate sample size, recognizing that this timeframe would preclude analysis of temporal trends but provide a robust snapshot of current disclosure practices. We excluded non-empirical publications (e.g., commentaries, letters, perspectives) both because we perceived the stakes to be higher for disclosing AI use in research than in commentaries and we anticipated that the conventional genre of empirical articles would support meaningful comparisons regarding disclosure content and location.

**Figure 1:**
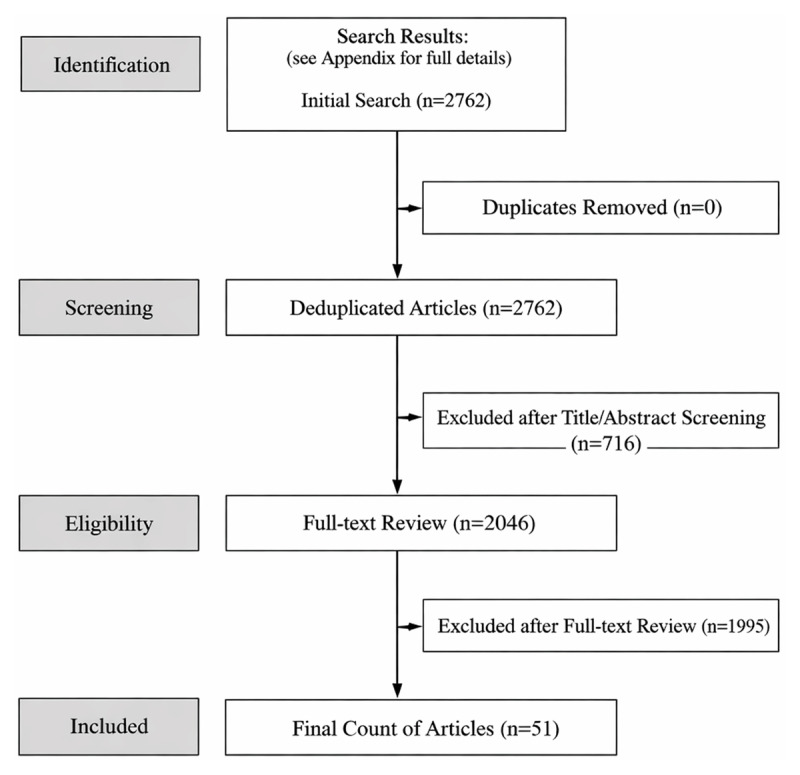
PRISMA Chart.

Screening and article selection were managed in *Covidence*, a web-based knowledge synthesis software. MA, HA, JC, and LM independently screened the titles and abstracts of all articles to exclude non-empirical publications. Full-texts of the remaining articles were then examined by MA and HA for AI-use disclosure statements. We classified articles as having an AI statement if they explicitly disclosed the use of an AI tool (e.g., ChatGPT) or described how AI was used in the preparation of the work. Articles that merely focused on, evaluated, or discussed an AI tool, without indicating that AI was used to generate or assist with the manuscript, were not counted as having an AI disclosure statement. For example, an article studying the effectiveness of AI in clinical reasoning, but lacking an explicit disclosure of AI involvement in the authorship process, would not be considered to have a statement. Articles with an AI-use disclosure statement proceeded to data extraction (Figure 1).

MA and HA each individually extracted data from approximately half of the articles using a structured Google Sheets template. They met regularly to ensure consistent interpretation, discussing ambiguous or complex disclosure statements and reaching consensus through discussion. For each article with an AI-use disclosure statement, we extracted:

- Bibliographic variables: DOI, article title, journal name, publication date, journal impact factor, publisher.
- Author-level variables (first author only): name, total publications, total citations, H-index, year of first publication, institutional affiliation, career position, and country of affiliation. These data were collected using Web of Science (WoS). Although we recognize that authors may have publications not indexed in the WoS, we viewed this as a reasonable proxy measure for publishing experience. We focused on first authors as they typically lead writing and analysis procedures. While we recognize that corresponding authors may also influence AI-disclosure, the first author focus ensured feasible study scope.

For bibliographic and author-level variables, in July 2025, we downloaded the metadata from WoS; if unavailable on WoS, we queried Scopus, except for author career position. Author career position was extracted from the article’s author information, or if unavailable, from ResearchGate. Additionally, based on a review of journal and publisher websites, we identified if AI reporting guidance was provided at the time of our study.

Disclosure-specific extractions focused on the name of AI tool(s) used, purpose of AI use, location of the disclosure statement (e.g., methods, acknowledgements, disclosures), verbatim text of the statement, statement word count, and the presence or absence of an attestation of responsibility (e.g., authors remain accountable for the content). We also extracted measures of transparency to situate AI disclosure statements within the broader context of established transparency practices. These included open access status, presence of a funding statement, conflict of interest disclosure, and data availability statement.

For analysis, we conducted descriptive statistics to characterize the bibliographic and author-level variables. To describe the content of AI-use disclosure statements, we conducted a descriptive content analysis [14] for AI tool used, the nature of usage, and any reference to responsibility attestation. Two researchers were involved in the analysis: each analyzed a subset of the disclosures, coming together regularly to discuss and resolve discrepancies.

## Results

Over the study period, 2,762 articles were published in the medical education journals sampled. Of these, we excluded 716 (25.9%) non-empirical articles. Of the remaining 2,046 articles, 2.5% (n = 51) had an AI disclosure statement (Appendix A) [15,16,17,18,19,20,21,22,23,24,25,26,27,28,29,30,31,32,33,34,35,36,37,38,39,40,41,42,43,44,45,46,47,48,49,50,51,52,53,54,55,56,57,58,59,60,61,62,63,64,65].

Thirteen of the journals included articles with disclosures. Of these, 12 (92.3%) provided author guidance on disclosure on their website/author instructions, or on their publisher’s website (Table 1). *BMC Medical Education* published nearly half the identified statements (n = 24, 47.1%), followed by *Medical Teacher* (n = 7, 13.7%) and the *Journal of Surgical Education* (n = 4, 7.8%). Nine journals did not include any articles with disclosures. Two journals (*African Journal of Health Professional Education* and *BMJ Stimulation & Technology Enhanced Learning*) did not publish any empirical research during the study period and, therefore, were not examined for AI-use disclosures.

**Table 1:** Distribution of AI-Use Disclosures by Journal.


JOURNAL	NO. ARTICLES PUBLISHED IN STUDY PERIOD	NO. EMPIRICAL ARTICLES	NO. OF ARTICLE WITH DISCLOSURES	PERCENTAGE OF ARTICLES WITH DISCLOSURES IN THE JOURNAL	AI DISCLOSURE GUIDANCE AVAILABLE

*Academic Medicine*	303	135	2	0.66%	Y

*Advances in Medical Education and Practice*	94	80	0	0.00%	Y

*Advances in Health Sciences Education*	81	63	1	1.23%	Y

*Anatomical Sciences Education*	87	59	2	2.30%	N

*BMC Medical Education*	856	796	24	2.80%	Y

*Canadian Medical Education Journal*	72	27	0	0.00%	Y

*Clinical Teacher*	137	97	1	0.73%	Y

*Focus on Health Professional Education*	8	3	2	25.00%	Y

*GMS Journal for Medical Education*	26	23	0	0.00%	N

*International Journal of Medical Education*	14	13	0	0.00%	N

*Journal of Continuing Education in the Health Professions*	41	30	1	2.44%	Y

*Journal of Educational Evaluation for Health Professions*	16	16	0	0.00%	Y

*Journal of Graduate Medical Education*	94	43	1	1.06%	Y

*Journal of Medical Education and Curricular Development*	78	68	0	0.00%	Y

*Journal of Surgical Education*	170	155	4	2.35%	Y

*Medical Education*	170	77	0	2.25%	Y

*Medical Education Online*	50	43	3	0.58%	Y

Medical Teacher	304	153	7	2.30%	Y

*Perspectives on Medical Education*	33	28	0	0.00%	Y

Simulation in Healthcare	42	30	0	0.00%	Y

Teaching and Learning in Medicine	62	44	1	1.61%	Y

Education for Health	24	14	2	6.25%	Y


**African Journal of Health Professional Education and BMJ Stimulation & Technology Enhanced Learning did not have any empirical studies published to analyze.

*Focus on Health Professional Education* had the highest proportion of disclosure statements (25%), although this reflects a small denominator of total articles published, with 2 of 8 containing disclosure statements. Across the other 23 journals, the prevalence of AI-use disclosure statements was low, with no journal exceeding 7% of published articles (Table 1).

### Author Characteristics

Authors were affiliated with institutions across all six World Health Organization (WHO) regions (Table 2), with 24 countries represented (Online Supplemental Appendix B). Fifty unique first authors from 46 institutions included disclosure statements in their articles. Authors affiliated with institutions in the Western-Pacific region accounted for the largest share of disclosures (n = 15, 29.4%), followed by Europe (n = 11, 21.6%) and the Americas (n = 11, 21.6%). Disclosures were also reported by authors from the Mediterranean (n = 9, 17.6%), Southeast Asia (n = 4, 7.8%), and Africa (n = 1, 2.0%). At the country level, USA, Germany, Iran and Australia were most represented.

**Table 2:** Regional Distribution (WHO Regions) of First Authors of Articles with AI-Use Disclosures (n = 51).


REGION	NUMBER OF ARTICLES (%)

Western Pacific (e.g., Australia, China, South Korea)	15 (29)

Americas (e.g., North, Central, South America)	11 (22)

Europe (e.g., Germany, Netherlands)	11 (22)

Eastern Mediterranean (e.g., Afghanistan, Egypt, Iran)	9 (18)

South-East Asia (e.g., Bhutan, Bangladesh, India)	4 (8)

Africa (e.g., South Africa)	1 (2)

Total	51 (100)


First authors had published a median of 11.5 publications (range 1–142, SD = 34.7). The median h-index, a citation-based metric, for authors was 4 (range 0–35, SD = 7.3) with the median author citations received being 55 (range 0–10,214, SD = 2266.2). Thirty-three (66.0%) authors published their first article within the last decade, and for 3 (6.0%) authors, this was their first publication.

Author career positions were variable. Among 51 first authors, there were 50 unique authors with the most common designation being Assistant Professor (n = 9, 17.6%), followed by: other (e.g. clinical roles or administrative positions) (n = 8, 15.7%), trainees or early career researchers (e.g. residents, PhD students, post-doctoral researchers) (n = 7, 13.7%), Associate Professor (n = 6, 11.8%), lecturers/senior lecturers (n = 6, 11.8%), research staff or fellows (n = 6, 11.8%), and Professor (n = 4, 7.8%). Four author career positions were unreported.

### AI tools and Nature of AI use

Authors disclosed the use of 18 unique AI tools. ChatGPT was the most disclosed tool (n = 19, 37.3%), followed by Otter.ai (n = 11, 21.6%) and Editage (n = 3, 5.9%) (Table 3). Five articles (9.8%) combined multiple tools, and 4 (7.8%) disclosed AI use without naming a specific tool. Four articles (7.8%) explicitly included negative disclosure statements, stating that no AI tools were used, such as: “*Generative AI was not used for any aspect of this study, including drafting of the manuscript*” [57]. The 4 negative disclosures originated from 2 authors in China, 1 in Hungary, and 1 in Qatar [45,46,57,60]. Negative disclosures were included because they represent a type of explicit disclosure practice and signal author awareness of disclosure expectations.

**Table 3:** AI Tools Reported in Disclosure Statements (n = 51).


AI TOOL	BRIEF DESCRIPTION OF TOOL	NUMBER OF ARTICLES (%)*

ChatGPT	Large Language Model	19 (37%)

Otter	AI-Powered Transcription Software	11 (22%)

Negative AI Disclosure	N/A	5 (10%)

Unspecified	N/A	4 (68%)

Editage	AI Editing for Researchers	3 (6%)

Claude	AI Assistant	2 (4%)

Curie	Clinical AI Assistant	2 (4%)

ASReview	AI tool for Systematic Reviews	1 (2%)

ClusterBot	AI systematic text categorization	1 (2%)

Consensus.app	AI-powered Search Engine	1 (2%)

Copilot	Generative AI assistant	2 (2%)

DeepL	Comprehensive AI Language Platform	1 (2%)

Grammarly	AI Writing Assistant	1 (2%)

Gemini	Large Language Model	1 (2%)

Hawk	Writing/reading AI Tool	1 (2%)

Open Evidence	Evidence-based clinical decision making AI tool	1 (2%)

POE AI Tools	AI Chatbot Aggregator	1 (2%)

RapidMiner	Data Analytics AI Tool	1 (2%)

Rev.com	Speech to Text Service	1 (2%)


*Some articles reported multiple tools hence the total is greater than 51.

We characterized the use of AI into six types based on the language in the disclosures (Table 4). These categories included: editing, transcription, thematic/data analysis, drafting and article screening. Use of AI for editing purposes was the most reported (n = 28, 54.9%), followed by transcription (n = 12, 23.5%), thematic/data analysis (n = 7, 13.7%), drafting (n = 3, 5.9%), and article screening in knowledge syntheses (n = 2, 3.9%).

**Table 4:** Nature of AI Use in Disclosure Statements (n = 51).


TYPE OF AI USE	DEFINITION	EXAMPLES	NUMBER OF ARTICLES (%)

Editing	Polishing	*“Claude v. 3.5 Sonnet has been used for language editing. All content and ideas remain the original work of the authors, with AI assistance to improve linguistic clarity.”*	28 (55)

Transcription	Text generation from audio/video	*“We used the Otter.ai transcribing software (Otter.ai Inc, California), with the RA reviewing each transcript for accuracy.”*	12 (24)

Analysis	Thematic and Statistical Analysis	*“The authors acknowledge availing of a language quality checker and editing tool that used Curie’s AI software freely available from Springer Nature webpage as mentioned in the submission guidelines. The authors also* *acknowledge the use of Claude 3.5 Sonnet software for thematic analysis of the qualitative data.”*	7 (14)

Drafting	Text generation	*“We acknowledge the assistance of the GPT-4 AI language model by OpenAI for support with statistical analysis and drafting of this manuscript”*	3 (6)

Literature Review: Article Screening	Includes article screening as a component of literature review	*“We used an open-source artificial intelligence (AI) tool, ASReview (V.1.4)* [21]*. ASReview employs a machine learning algorithm that prioritizes articles based on their textual proximity to previously identified relevant articles (by the researchers). The tool consequently reduces the time and effort required during the initial screening phase but does not replace the initial screening of articles by researchers.”*	2 (4)


**Some articles cited multiple uses of AI hence the total being greater than 49.

AI was most frequently described as being used for language-related functions. Editing was cited in 28 (54.9%) articles, with representative phrasings such as “*we would like to thank Editage for English language editing*” [39] *or* “*the authors used ChatGPT to improve language and readability*” [41]. Transcription was described in 12 (23.5%) articles: e.g., “*the interview data was transcribed using Otter.ai and then manually checked for accuracy*” [61].

Eight unique articles (15.7%) disclosed more substantive AI uses. For example, 3 articles (5.8%) disclosed use of thematic analysis (e.g., one group of “*authors acknowledge the use of Claude 3.5 Sonnett for thematic analysis of qualitative data*”) [14]. AI use for data analysis was reported in another 3 (5.8%) articles, described in one study as “*ChatGPT was used to assist in identifying descriptive trends and generating narrative summaries*” [63]. One article (1.9%) described both thematic and data analysis. Two articles (3.9%) reported AI for screening purposes, with one article saying, “*We used an open-source artificial intelligence (AI) tool, ASReview (V.1.4)… ASReview employs a machine learning algorithm that prioritizes articles based on their textual proximity to previously identified relevant articles (by the researchers)*” [20].

Disclosure statements were typically concise, a single sentence averaging 43 words (Range 11–200, SD 36.1). For instance, one stated that “*DeeplL AI writing assistant was used to enhance this manuscript for methodology, clarity, and style*” [16], while another reported that “*Chat-GPT 3 was used to improve the clarity of the manuscript and for language editing*” [37]. As these representative examples illustrate, disclosures tended to include general terms such as “enhance” or “improve” without detailing the particular characteristics that were enhanced or how the AI was used to achieve such enhancement. In contrast, only 2 articles (3.9%) had a disclosure word count of over 100 words and offered more precise indications of the nature of AI use. For example, one disclosure stated:

“*The questionnaire for this study was designed with the assistance of AI tools, demonstrating AI’s potential as a research collaborator. The collected data were initially explored using ChatGPT, an AI-powered language model, to assist in identifying descriptive trends and generating narrative summaries. ChatGPT was not used for statistical calculations or hypothesis testing. Artificial Intelligence tool, specifically OpenAI’s ChatGPT (version GPT-4, accessed via ChatGPT Plus) we used at various stages of this research. The following contributions were made: Questionnaire Development: ChatGPT was used to draft survey items aligned with the research objectives. Prompts included, for example: “Design a student survey to evaluate the use of AI tools in medical education across academic, clinical, and research contexts.” Thematic Analysis Assistance: For qualitative responses, ChatGPT helped group answers into initial themes. These were reviewed, corrected, and finalized by the authors to ensure accuracy and context. Narrative Drafting: ChatGPT was used to generate narrative summaries of the findings and to draft portions of the introduction, results, and discussion. Prompts included: “Summarize Likert-scale findings and interpret trends,” and “Rewrite this paragraph in an academic tone.” Language Polishing: ChatGPT helped improve the clarity, grammar, and coherence of the manuscript*” [63].

### Attestation of Responsibility

Only 26% (n = 13) of disclosures included an attestation of responsibility. For example, authors wrote “*After using this tool, the authors reviewed and edited the content as needed and took full responsibility for the content of the publication*” [24]. Attestation statements were most commonly included when disclosures appeared in a dedicated AI disclosure section (n = 10, 76.9%), or in the acknowledgements (n = 3, 23.1%), such as: “*During the preparation of this manuscript, the authors utilized ChatGPT to help refine the language. After using this tool, the authors reviewed and made any necessary adjustments and took full responsibility for the content of the publication*” [57]. By contrast, these attestation statements were absent when the disclosures appeared in the methods (n = 0). Attestation statements highlighted retrospective human oversight of and responsibility for AI use. Typical attestation phrasing included “*reviewed and edited the content as needed*”, “*made any necessary adjustments*”, and “*took full responsibility for the content of the publication*” [24,31,48,57]. No disclosures with attestations described concrete validation or verification processes; they were written as generalized declarations of accountability rather than specific descriptions of oversight or verification practices.

### Location of Disclosure Statements

Placement of disclosure statements varied. Approximately one-third of the disclosures (n = 19, 37.3%) appeared in the acknowledgements section. Another 15 (29.4%) articles appeared in a dedicated AI-use disclosure section, which consistently appeared at the end of the published manuscript, grouped with other declarations such as conflict of interest or data availability statements. For the remaining statements, 14 (27.5%) articles exclusively reported their disclosure in the methods, 2 (3.9%%) in both the methods and a dedicated AI disclosure section, and 1 (2%) in the discussion. In relation to the location and content of disclosures, based on disclosure counts, we noted that those located in the methods primarily described AI use for transcription, e.g., “*All the audio-recorded interviews were transcribed verbatim into Microsoft Office using Otter.ai*” [38].

### Transparency Metrics

We found that 42 articles (82.4%) were published as open-access, 42 articles (82.4%) included a funding statement, 40 articles (78.4%) had a conflict-of-interest statement, and 37 articles (72.5%) had a data availability statement. In most cases, AI-use disclosures appeared directly alongside these established measures of academic transparency (n = 34, 66.7%) at the end of the publication.

## Discussion

Our findings highlight three primary issues for consideration in our field: the low rate of disclosure, the geographical pattern of disclosures concentrated in non-native English-speaking regions, and the practice of superficial, safe disclosures.

Our analysis suggests that AI-use disclosure is rare in medical education: only 2.5% of empirical articles published in 24 medical education journals in the first half of 2025 included a disclosure. This corroborates medical education journal editors’ subjective experience of infrequently seeing disclosures in submitted manuscripts [66]. It also resembles bibliometric results from other research domains: e.g., an analysis of academic radiology articles in 2024 found that 34 of 1998 manuscripts (1.7%) disclosed AI use [67]. The low rate could be interpreted in many ways. It may reflect low AI use. It may signal lack of awareness of AI use, such as when researchers use a tool not knowing that it is powered by AI. Or it may reflect a tendency towards nondisclosure of AI use. Some evidence suggests the last [68]: for instance, since 2023, analyses by “integrity specialists” have flagged hundreds of published research papers with obvious signs of undisclosed AI use, such as those that contain the phrase “regenerate response” [69]. Given that the field of medical education research is not immune to undisclosed AI use [70], we need to explore the underpinning influences on our field’s current rate of AI-use disclosure.

One influence is the lack of clarity. Not only are journal policies on AI use and disclosure shifting quickly due to the fast-changing AI landscape, journals’ positions vary. For authors submitting, and resubmitting, their manuscripts, disclosure requirements may appear murky and complicated. Another influence is the divergent attitudes among researchers about AI use and disclosure: surveys have clearly shown that we don’t agree about what AI should be used for and when those uses require disclosure [8,11]. A third influence is psychological/cultural and institutional barriers that make researchers hesitant to disclose AI use to improve their writing. They may experience a sense of “demonization” of AI [71], inducing guilt or shame about using it to support their work [72] and fear of consequences such as academic stigma and evaluation bias [73]. These concerns have recently also surfaced in relation to research teams [74], where decisions about AI use and disclosure may be implicit, unevenly negotiated, or avoided altogether. Such dynamics can negatively impact team functioning, strain trust, and complicate authorship negotiations. Finally, the lack of published disclosures may act like a self-fulfilling prophecy: if we suspect others are using AI but we rarely see AI-use disclosures, that can send a tacit message that disclosure is unnecessary.

Based on frequency, our findings also demonstrate a particular geographical pattern to AI-use disclosure in the analyzed articles, with most authored by individuals from non-native English-speaking regions of the world. This likely explains the dominant use of AI tools for language-related tasks, like editing, translating and grammar checking, all of which are valuable to authors writing in English as a non-native language, and resonates with other studies that have reported higher AI use for writing support by non-native English researchers [75]. Given this finding, medical education needs to be aware of the possibility of stigmatization, given that non-native English academic writing is more likely to raise reviewers’ suspicions about AI use [76], and is also known to be prone to misclassification by GPT detectors [77]. The four negative disclosures in our dataset all came from authors in non-native English-speaking regions, which may suggest an attempt to deflect such potential stigmatization. Medical educators need to be aware that, rather than levelling the global playing field in academic publishing, AI-use disclosure might intensify inequity and bias against authors from some parts of the world [78], further exacerbating the global North dominance in the field’s literature [79,80]. As a recent AMEE Guide cautioned, the equity implications of AI-use disclosure practices and policies require ongoing attention [81].

The disclosures we analyzed were almost uniformly brief, describing general uses such as “editing”, and lacking in detail that would allow a reader to understand precisely how the author engaged with the AI, iteratively refined and structured its work, and verified its outputs. We interpret this as “safe” disclosure practice: authors are mostly including the required elements of tool, task, and attestation, but rarely disclosing substantive, intellectual tasks, or elaborating the ‘how’ of their interaction with the AI, including specific verification practices underpinning their attestations of responsibility. On the one hand, this might be good news: the disclosed uses we found mostly map onto the “acceptable” uses outlined in a recent framework distinguishing ethically acceptable, contingent and suspect uses of AI [82]. On the other hand, though, we worry that our dataset includes few disclosures in the framework’s “contingent” or “suspect” ranges, not because researchers are not using AI in these ways, but because they perceive themselves to be on shaky ground in these areas. While our study was not designed to judge the accuracy of the AI-use disclosures we analyzed, the predominance of “safe disclosures” suggests that future research should explore how authors are deciding what and how to disclose.

The combination of low disclosure rates and safe disclosure practice produces a “transparency paradox” [73], in which mandatory disclosure that does not attend to social complexities leads to both nondisclosed AI use, and disclosure “theatre” in which published disclosures are more performative than informative [83]. One possible solution is for journals to create dropdown menus of possible AI uses and verification strategies, signaling sanctioned uses and expected levels of detail. These menus might draw from emerging disclosure frameworks specifying tiers of usage [84] or using CREDiT-like author contribution frameworks [85]. Another solution might be to shift away from ‘disclosure’ language altogether and make AI use a routine part of methods reporting, as recommended in the recent AMEE Guide [86]. This could help to normalize writing about AI use in manuscripts, thus reducing the negative valence associated with disclosure in the current literature. Given our results, however, we anticipate that authors will need explicit guidance to include responsibility attestations when reporting AI use within their Methods.

### Limitations

This study has several limitations. First, our analysis was limited to the first 6 months of 2025. Because editors have reported a recent increase in AI-related disclosures [65], it is possible that disclosure rates were higher during this period, but we lack data to examine changes or trends over time. Moreover, it is possible that the low frequency of disclosures we observed may reflect the field’s publication timelines, given an average lag of approximately 188 days from submission to publication [86], suggesting that the manuscripts referenced by editors may not yet have been published. Notwithstanding these temporal characteristics, our dataset is well within the Cochrane Collaboration standards for timeliness [87]. Second, we did not compare articles with AI disclosures to those without, which limits our ability to assess differences in overall transparency practices. Third, although we reviewed each journal’s AI instructions at the time of data collection, it is possible that those instructions differed from the guidance in place when authors were conducting and publishing their work. That said, many journals draw on shared frameworks such as COPE guidance [88], which has remained relatively stable over this period, making substantial differences in expectations less likely. Finally, because many journals impose word limits, authors may have a limited word count to include and/or elaborate on their AI use, potentially constraining the detail and desire to include a disclosure statement.

## Conclusion

AI-use disclosures in medical education journals are rare and appear mostly in work from non-native English-speaking regions of the world. A shared disclosure practice is evident: name the tool and affirm author responsibility, but describe use superficially. This suggests a practice of “safe” disclosure that may fail to satisfy the goal of ensuring transparent and ethical AI use in research.

## Additional File

The additional file for this article can be found as follows:

10.5334/pme.2431.s1 Appendix A. Frequency of AI-Use Disclosure Statements by Countries.

10.5334/pme.2431.s2 Appendix B. World Health Organization Regional Classification.

## Preprint

This work has been submitted as a preprint to medRxiv.
