# Supplementary material for: The Presence and Nature of AI-Use Disclosure Statements in Medical Education Journals: A Bibliometric Study
Source: Perspect Med Educ. 2026 Mar 5;15(1):212–25. doi: 10.5334/pme.2431 (PMC12962250; doi:10.5334/pme.2431)
Supplement: Appendix A. — Frequency of AI-Use Disclosure Statements by Countries. [file pme-15-1-2431-s1.pdf]

The Presence and Nature of AI-Use Disclosure Statements in Medical Education Journals: A bibliometric study

Appendix A – Frequency of AI-Use Disclosure Statements by Countries

Country	Frequency
United States of America	8
Germany	6
Iran	5
Australia	5
Japan	3
China	3
Nepal	2
New Zealand	2
Saudi Arabia	1
Netherlands	1
Brazil	1
Canada	1
South Africa	1
Turkey	1
Bangladesh	1
Thailand	1
Hungary	1
Singapore	1
England	1
Pakistan	1
Palestine	1
Uruguay	1
Egypt	1
Qatar	1
