# Supplementary material for: The Presence and Nature of AI-Use Disclosure Statements in Medical Education Journals: A Bibliometric Study
Source: Perspect Med Educ. 2026 Mar 5;15(1):212–25. doi: 10.5334/pme.2431 (PMC12962250; doi:10.5334/pme.2431)
Supplement: Appendix B. — World Health Organization Regional Classification. [file pme-15-1-2431-s2.pdf]

Supplementary Material

Appendix B. World Health Organization Regional Classification

Author institutional affiliations were categorized according to the regional classification framework established by the World Health Organization (WHO). WHO divides Member States into six geographic regions as outlined below:

1. African Region (AFR)
2. Region of the Americas (AMR)
3. South-East Asia Region (SEAR)
4. European Region (EUR)
5. Eastern Mediterranean Region (EMR)
6. Western Pacific Region (WPR)

Regional categorization was applied at the country level based on official WHO designations at the time of data extraction.
